# Comparative Study of Premixed Versus Sequential Administration of Hyperbaric Bupivacaine and Fentanyl for Subarachnoid Block in Caesarean Section: A Randomized Controlled Trial

**DOI:** 10.7759/cureus.97375

**Published:** 2025-11-20

**Authors:** Rahul Kalshan, Ranju Gandhi, Trisha Nidhi, Apoorva Singh

**Affiliations:** 1 Anaesthesia and Intensive Care, Vardhman Mahavir Medical College (VMMC) and Safdarjung Hospital, New Delhi, IND

**Keywords:** caesarean section, fentanyl, hyperbaric bupivacaine, maternal hypotension, sequential injection, spinal anaesthesia

## Abstract

Background: Spinal anaesthesia is the standard technique for elective caesarean section due to its simplicity, rapid onset, and maternal safety profile. While combining hyperbaric bupivacaine with intrathecal fentanyl enhances anaesthetic efficacy, the sequential drug administration may influence the spread, block dynamics, and haemodynamic stability.

Methods: In this prospective, randomised controlled study, 180 ASA I-II parturients were allocated to either Group M (premixed bupivacaine 9 mg + fentanyl 15 mcg) or Group S (sequential administration of the same drugs). Primary outcomes were time to achieve T5 sensory level, duration of sensory and motor blocks, and incidence of hypotension. Secondary outcomes included vasopressor use, Apgar scores, and umbilical cord blood gas analysis.

Results: Group S achieved T5 sensory level significantly faster than Group M (2.88 ± 2.22 vs. 6.04 ± 2.59 min; P = 0.0001). The duration of sensory block was longer in Group S (151.59 ± 22.46 min vs. 123.49 ± 22.64 min; P = 0.0001). Incidence of hypotension was lower in Group S (1.1%) compared to Group M (2.2%) (P = 0.563). Phenylephrine use was also reduced in Group S (P = 0.563). Apgar scores and umbilical cord blood gases were comparable between groups (P > 0.05).

Conclusion: Sequential intrathecal administration of hyperbaric bupivacaine and fentanyl provides superior sensory block dynamics and improved haemodynamic stability compared to premixed administration, without compromising neonatal outcomes. This technique is simple, effective, and suitable for routine obstetric anaesthesia.

## Introduction

Spinal anaesthesia is the preferred technique for elective caesarean section due to its rapid onset, simplicity, and favourable safety profile for both the mother and fetus [1,2]. Compared with general anaesthesia, it is associated with reduced maternal and neonatal morbidities [2]. However, one of the most common and clinically significant complications is maternal hypotension, which can result in nausea, vomiting, dizziness, decreased uteroplacental perfusion, and fetal acidosis [3,4].

To mitigate this, intrathecal opioids such as fentanyl are commonly combined with local anaesthetics like hyperbaric bupivacaine. This combination improves the quality of sensory block, extends analgesia, and reduces the required dose of local anaesthetic [5,6]. When premixed, however, the solution's baricity may be altered, potentially leading to unpredictable spread in cerebrospinal fluid (CSF) and variable haemodynamic responses [7,8].

An alternative is sequential intrathecal administration, where hyperbaric bupivacaine is injected first, followed by fentanyl through a separate syringe. This approach maintains each drug’s baricity and may facilitate a more predictable block, with staggered sympathetic blockade and improved haemodynamic stability [9,10].

Although studies have explored this technique, findings remain limited and inconsistent. This randomised controlled trial was conducted to compare premixed versus sequential intrathecal administration of hyperbaric bupivacaine and fentanyl in patients undergoing elective caesarean section. We hypothesised that the sequential method would result in faster onset of sensory block, improved haemodynamic profile, and comparable neonatal outcomes.

## Materials and methods

Study design and ethical approval

This prospective, randomised, interventional trial was conducted at the Department of Anaesthesia and Intensive Care, Vardhaman Mahavir Medical College and Safdarjung Hospital, New Delhi, between October 31, 2017, and March 31, 2019. Ethical approval was obtained from the Institutional Ethics Committee (30/10/2017), and the trial was prospectively registered with the Clinical Trials Registry-India (CTRI/REF/2018/03/017788). Written informed consent was obtained from all participants. The study adhered to the Declaration of Helsinki (2013).

Participants

A total of 180 parturients aged 18-35 years, with ASA Physical Status I-II and scheduled for elective caesarean section, were enrolled. Inclusion criteria were as follows: height 145-165 cm and BMI < 35 kg/m². Exclusion criteria included coagulopathy, infection at the puncture site, known allergy to study drugs, pre-existing hypertension, and obstetric complications.

Randomisation and allocation

Participants were randomised into two groups (n = 90 each) using a computer-generated sequence. Simple randomisation without stratification or blocking was used. The allocation sequence was maintained with sealed opaque envelopes prepared by an independent investigator who was not blinded; however, the data collector and outcome assessor were blinded to group assignment. The groups allocated were Group M (mixture), who received 1.8 mL of 0.5% hyperbaric bupivacaine (9 mg) premixed with 0.3 mL fentanyl (15 mcg) in a single syringe, and Group S (sequential) who received 1.8 mL of hyperbaric bupivacaine (9 mg) followed by 0.3 mL fentanyl (15 mcg) injected sequentially through the same puncture using two syringes.

Anaesthesia protocol

All patients were premedicated with intravenous (IV) ranitidine 50 mg and metoclopramide 10 mg. Standard monitors (ECG, non-invasive blood pressure, SpO_²_) were applied. Preloading was performed with 10 mL/kg of Ringer’s lactate. Subarachnoid block was performed at L3-L4, with a 25 G Quincke needle under aseptic conditions. Drugs were injected at 0.1 mL/s, and patients were positioned supine, with a 15° left tilt immediately after injection to reduce aortocaval compression [7,8].

Monitoring and outcome assessment

Haemodynamic variables (heart rate (HR), systolic blood pressure (SBP), diastolic blood pressure (DBP), mean arterial pressure (MAP), SpO₂) were recorded at baseline, immediately after injection, at one, three, and five minutes and every five minutes thereafter until delivery. Hypotension was defined as >20% decrease in MAP from baseline and treated with 50 mcg phenylephrine IV. Bradycardia (HR < 50 bpm) was treated with 0.5 mg atropine IV.

Block assessment was performed bilaterally using pinprick and alcohol swab for sensory block, and the Modified Bromage Scale for motor block.

The primary outcomes were time to T5 sensory level, duration of sensory and motor block, and incidence of hypotension, while the secondary outcomes were total phenylephrine requirement, Apgar scores at one and five minutes, umbilical cord blood gas parameters, and maternal side effects (nausea, vomiting, pruritus, shivering). The patients were followed postoperatively for regression of sensory and motor block, time to first analgesic request, and maternal satisfaction. No changes were made to the pre-specified primary or secondary outcomes after the trial commenced.

Sample size calculation

The sample size was calculated using the formula described by Keera and Elnabtity [11], with an expected difference in the incidence of hypotension between groups. At 80% power and α = 0.05, 82 subjects per group were required. To account for dropouts, 90 patients were recruited per group.

Statistical analysis

Data were analyzed using IBM SPSS Statistics for Windows, Version 22.0 (Released 2013; IBM Corp., Armonk, NY, USA). Continuous variables were expressed as mean ± SD and compared using Student’s t-test or Mann-Whitney U test as appropriate. Categorical variables were analyzed using chi-square or Fisher’s exact test. A P-value of less than 0.05 was considered statistically significant.

## Results

**Figure 1:**
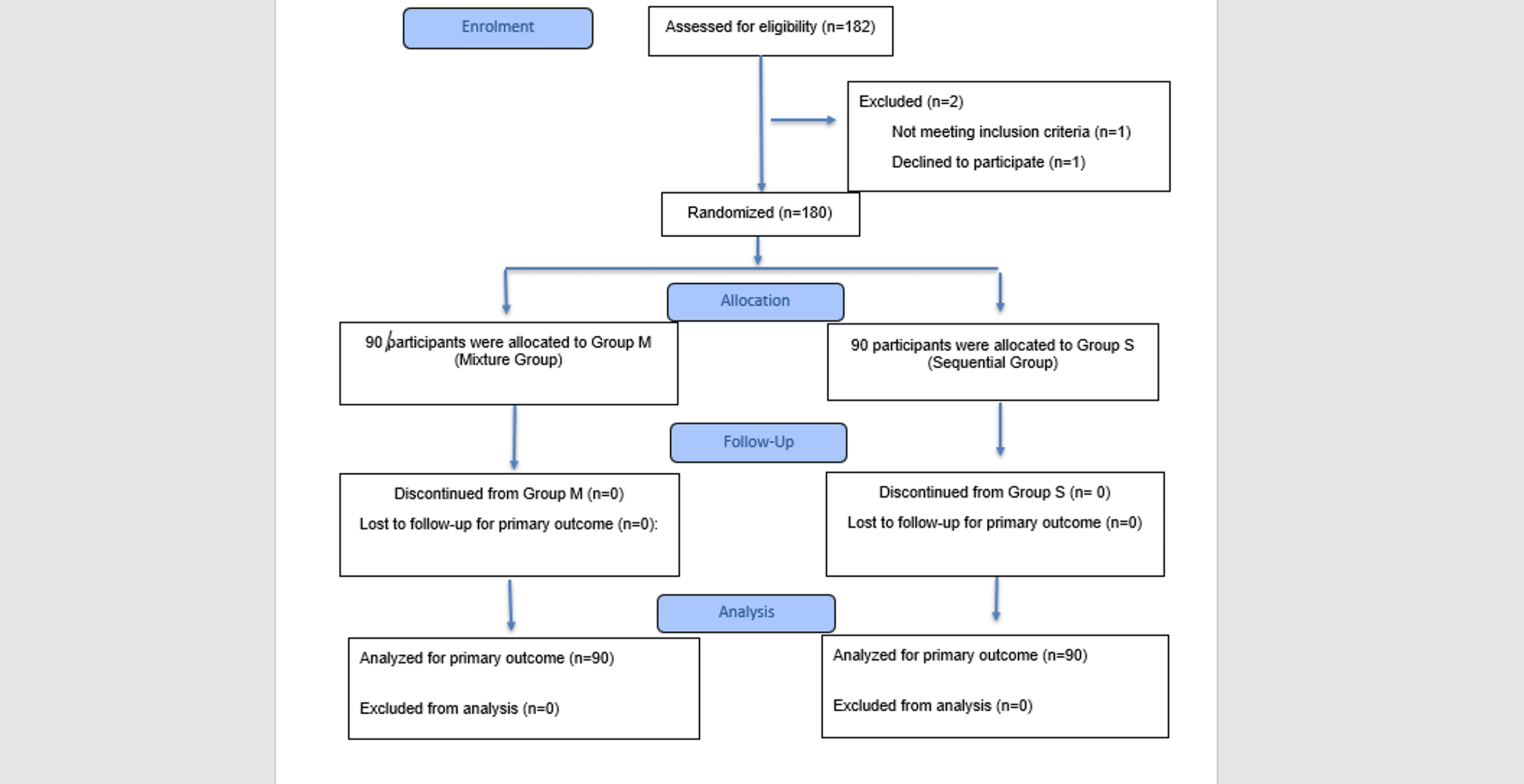
CONSORT flow diagram of the study participants Progress of patients through enrolment, allocation, follow-up, and analysis. Reasons for exclusion included non-eligibility (outside BMI/height range), refusal to consent, or contraindication to spinal anaesthesia.

Of the 182 patients enrolled, two patients were excluded from the study, and the remaining 180 patients were included in the final analysis (Figure 1).

Demographic and baseline characteristics

The demographic and baseline characteristics of the study participants in both groups are given in Table 1. Data are presented as mean ± SD. No significant differences were observed in age, height, weight, BMI, gestational age, or duration of surgery between groups (P > 0.05).

**Table 1:** Demographic and baseline characteristics of the study participants Data are represented as mean ± standard deviation. Group M (mixture group): Received 1.8 mL of 0.5% hyperbaric bupivacaine (9 mg) premixed with 0.3 mL fentanyl (15 mcg) as a single injection intrathecally. Group S (sequential group): Received 1.8 mL of 0.5% hyperbaric bupivacaine followed by 0.3 mL fentanyl in two syringes, injected sequentially at the same spinal puncture.

Variable	Group M (n = 90)	Group S (n = 90)	P-value
Age (years)	27.4 ± 3.2	26.9 ± 3.5	0.291
Height (cm)	156.2 ± 5.1	157.0 ± 4.8	0.402
Weight (kg)	64.7 ± 5.3	63.9 ± 6.0	0.309
BMI (kg/m²)	26.5 ± 1.2	26.3 ± 1.4	0.416
Gestational age (weeks)	38.4 ± 0.7	38.5 ± 0.8	0.637
Duration of surgery (min)	49.5 ± 7.6	50.1 ± 6.9	0.483

Block characteristics

The mean time to achieve T5 sensory level was significantly shorter in Group S compared with Group M (2.88 ± 2.22 vs. 6.04 ± 2.59 minutes; P < 0.001) (Table 2). The duration of sensory block was significantly longer in Group S (151.6 ± 22.5 minutes) than in Group M (123.5 ± 22.6 minutes; P < 0.001). Motor block duration followed a similar pattern with Group S, showing significantly prolonged block (P < 0.001). The highest level of sensory block achieved was higher in Group S, with 59% reaching T4 and 2% reaching T3 compared with 13% and 0% in Group M, respectively (P < 0.001).

**Table 2:** Block characteristics and haemodynamic changes with their adverse effects, including nausea, vomiting, pruritus, and shivering in the two groups Values are presented as mean ± standard deviation or percentages. T5: fifth thoracic dermatome, T4: fourth dermatome, T3: third thoracic dermatome. Group M (mixture group): Received 1.8 mL of 0.5% hyperbaric bupivacaine (9 mg) premixed with 0.3 mL fentanyl (15 mcg) as a single injection intrathecally. Group S (sequential group): Received 1.8 mL of 0.5% hyperbaric bupivacaine followed by 0.3 mL fentanyl in two syringes, injected sequentially at the same spinal puncture.

Parameter	Group M (n = 90)	Group S (n = 90)	P-value
Time to T5 sensory level (minutes)	6.04 ± 2.59	2.88 ± 2.22	<0.001
Duration of sensory block (minutes)	123.49 ± 22.64	151.59 ± 22.46	<0.001
Duration of motor block (minutes)	112.45 ± 19.32	140.11 ± 20.28	<0.001
Maximum sensory level (T4/T3)	13%/0%	59%/2%	<0.001
Hypotension incidence (%)	2.2%	1.1%	0.563
Phenylephrine dose (µg)	89.5 ± 18	58.7 ± 15	0.563
Nausea	12.2%	10.0%	0.621
Vomiting	4.4%	3.3%	0.715
Pruritus	8.9%	7.8%	0.798
Shivering	10.0%	6.7%	0.471

Haemodynamic stability and adverse effects

The incidence of hypotension was lower in Group S (1.1%) than in Group M (2.2%), though this difference was not statistically significant (P = 0.563). Phenylephrine requirement was also lower in Group S (58.7 ± 15 mcg) compared with Group M (89.5 ± 18 mcg) without reaching statistical significance (P = 0.563). Incidents of nausea, vomiting, pruritus, and shivering were comparable. Hence, Group S demonstrated a trend of lower incidence of hypotension and reduced vasopressor requirement, but differences were not statistically significant. Data are presented as mean ± SD or percentages.

Neonatal outcomes

Neonatal Apgar scores at one and five minutes were similar between the two groups (Table 3). Umbilical cord blood gas analyses, including pH, pO₂, pCO₂, and bicarbonate, were also comparable. No adverse neonatal outcomes were observed. Apgar scores and cord gas values did not differ significantly between groups (P > 0.05). Data are presented as mean ± SD or percentages.

**Table 3:** Neonatal outcomes in the two groups Values are presented as mean ± standard deviation. Apgar: appearance, pulse, grimace, activity, respiration, pO₂: partial pressure of oxygen, pCO₂: partial pressure of carbon dioxide, HCO₃⁻: bicarbonate, n: no. of subjects. Group M (mixture group): Received 1.8 mL of 0.5% hyperbaric bupivacaine (9 mg) premixed with 0.3 mL fentanyl (15 mcg) as a single injection intrathecally. Group S (sequential group): Received 1.8 mL of 0.5% hyperbaric bupivacaine followed by 0.3 mL fentanyl in two syringes, injected sequentially at the same spinal puncture.

Variable	Group M (n = 90)	Group S (n = 90)	P-value
Apgar score at one minute	8.8 ± 0.5	8.9 ± 0.4	0.221
Apgar score at five minutes	9.8 ± 0.4	9.9 ± 0.3	0.145
Umbilical pH	7.28 ± 0.04	7.29 ± 0.05	0.302
Umbilical pO₂ (mmHg)	25.6 ± 2.1	25.8 ± 2.3	0.448
Umbilical pCO₂ (mmHg)	48.1 ± 2.6	47.9 ± 2.5	0.583
Umbilical HCO₃⁻ (mmol/L)	20.6 ± 1.4	20.7 ± 1.3	0.495

**Figure 2:**
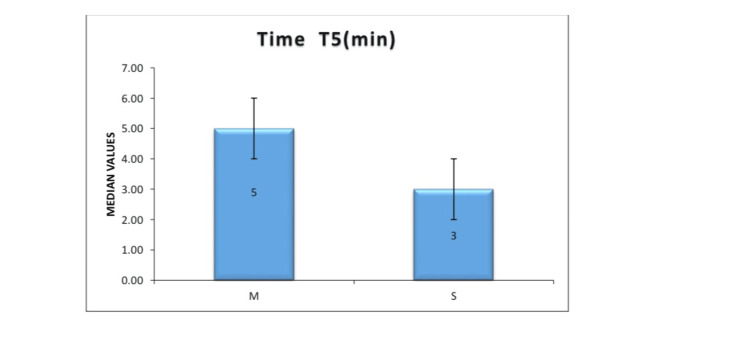
Time to achieve T5 sensory level in Group M (mixture) and Group S (sequential) Group S demonstrated a significantly faster onset of sensory (2.88 ± 2.22 minutes; P < 0.001). T5: fifth thoracic dermatome. Group M (mixture group): Received 1.8 mL of 0.5% hyperbaric bupivacaine (9 mg) premixed with 0.3 mL fentanyl (15 mcg) as a single injection intrathecally. Group S (sequential group): Received 1.8 mL of 0.5% hyperbaric bupivacaine followed by 0.3 mL fentanyl in two syringes, injected sequentially at the same spinal puncture.

Figure 2 depicts the time to achieve a T5 sensory block. Group S showed significantly faster onset than Group M (P < 0.001).

**Figure 3:**
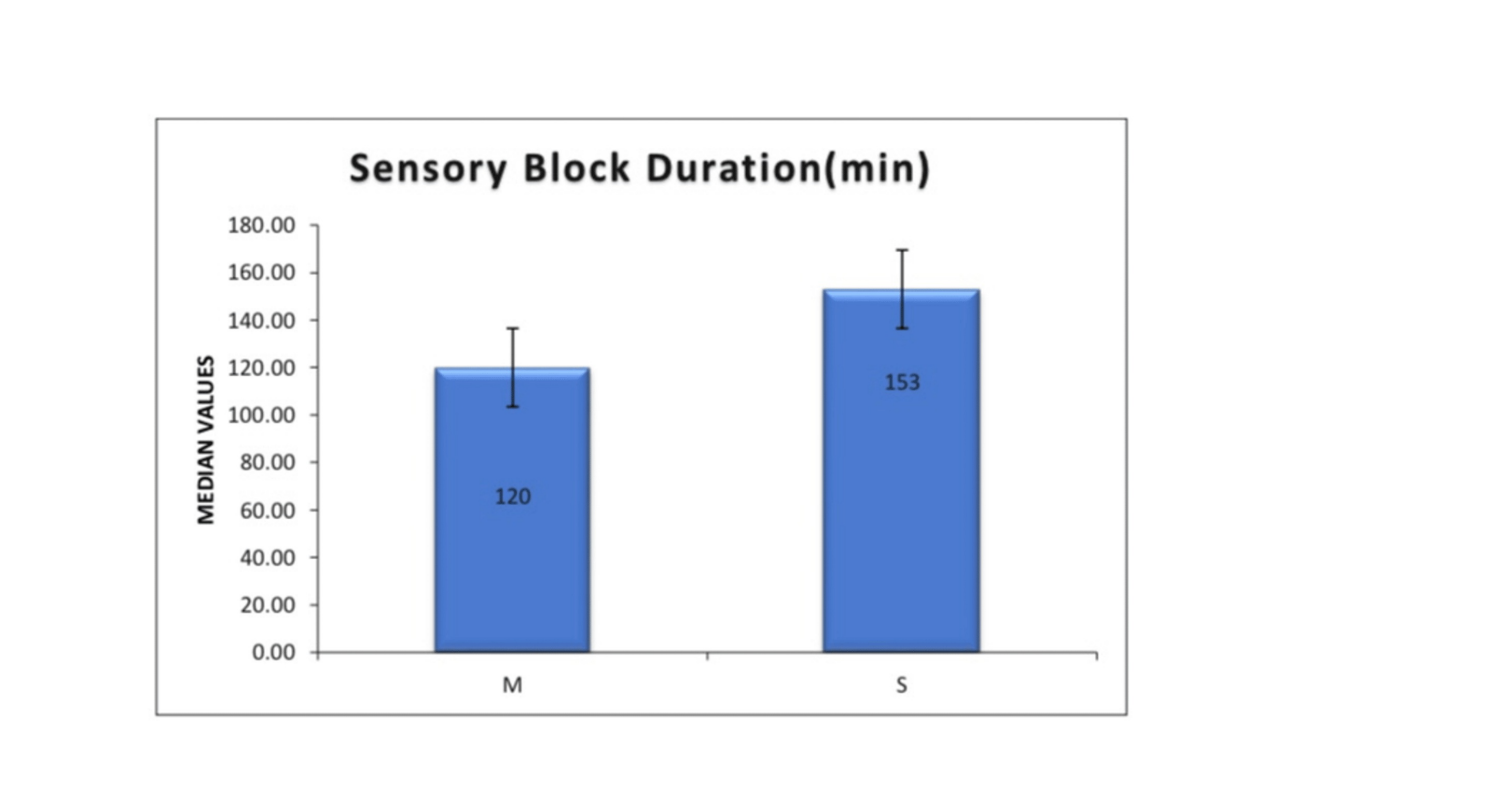
Duration of sensory block in Group M and Group S Group S exhibited a longer duration of sensory blockade compared to Group M (151.6 ± 22.5 minutes vs. 123.5 ± 22.6 minutes; P < 0.001). Group M (mixture group): Received 1.8 mL of 0.5% hyperbaric bupivacaine (9 mg) premixed with 0.3 mL fentanyl (15 mcg) as a single injection intrathecally. Group S (sequential group): Received 1.8 mL of 0.5% hyperbaric bupivacaine followed by 0.3 mL fentanyl in two syringes, injected sequentially at the same spinal puncture.

Figure 3 depicts the duration of the sensory block. Group S exhibited longer block duration (P < 0.001).

**Figure 4:**
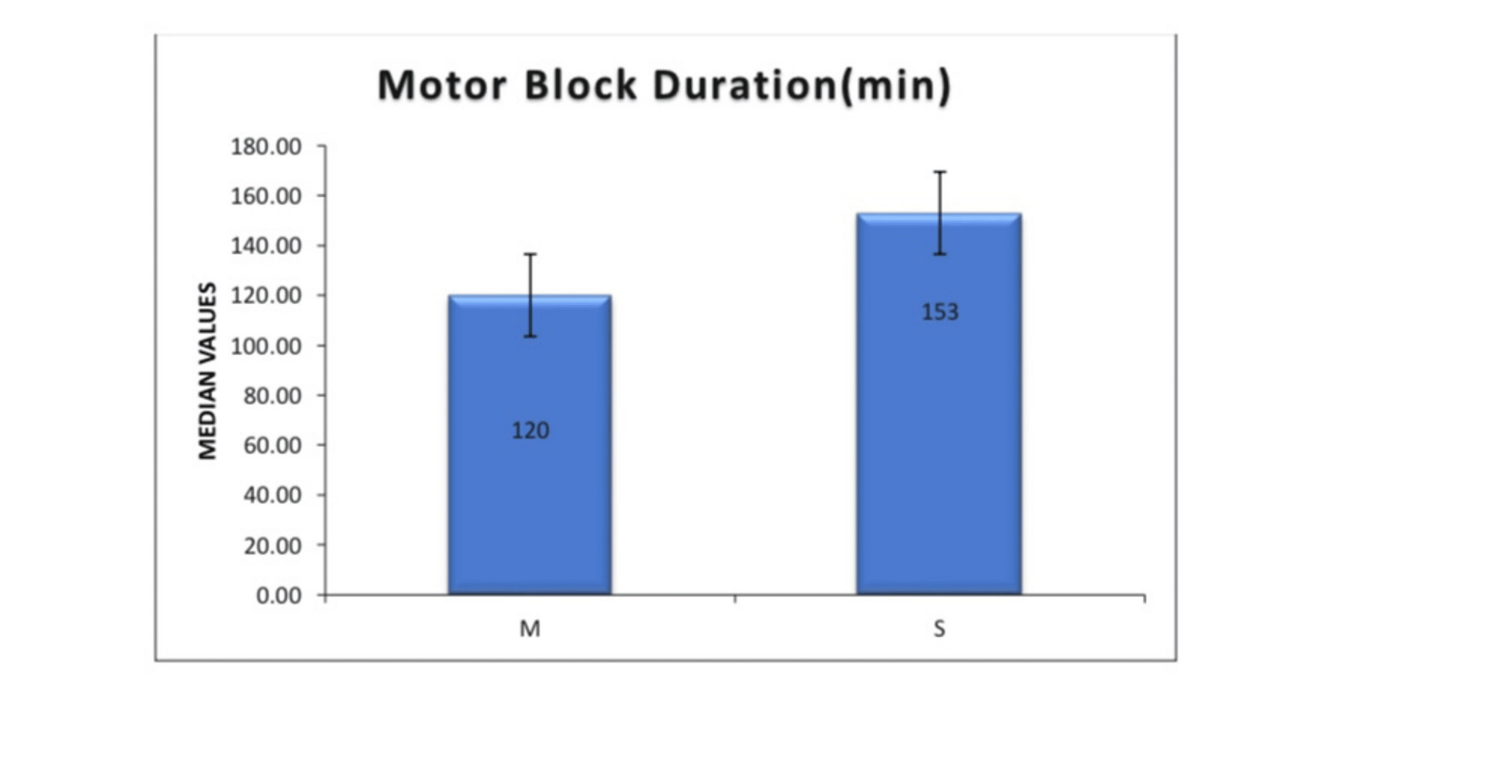
Duration of motor block in Group M and Group S Sequential injection given in Group S led to a statistically significant prolongation of motor blockade compared to injection given in mixture form (140.11 ± 20.28 minutes vs. 112.45 ± 19.32 minutes; P < 0.001). Group M (mixture group): Received 1.8 mL of 0.5% hyperbaric bupivacaine (9 mg) premixed with 0.3 mL fentanyl (15 mcg) as a single injection intrathecally. Group S (sequential group): Received 1.8 mL of 0.5% hyperbaric bupivacaine followed by 0.3 mL fentanyl in two syringes, injected sequentially at the same spinal puncture.

Figure 4 depicts the duration of motor block. Sequential injection significantly prolonged motor block (P < 0.001).

**Figure 5:**
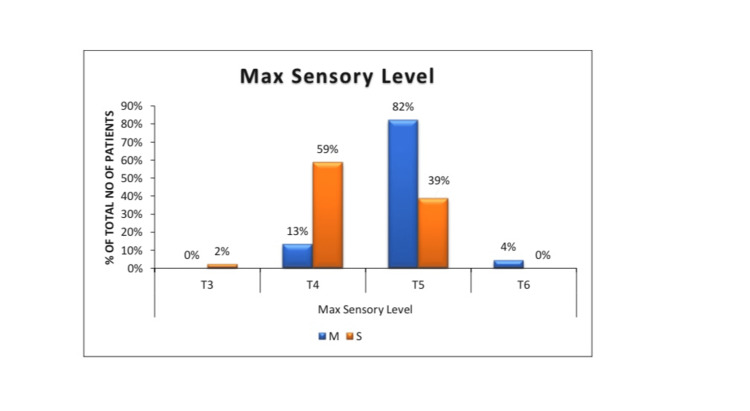
Maximum sensory level achieved in both groups Of the patients, 59% reached T4 level and 2% reached T3 level when the drugs were given sequentially, compared to 13% reaching T4 level and 0% reaching T3 level when the drugs were given in a mixture form. Hence, sequential administration of drugs reached higher dermatomes (T4 or above) compared to the administration of a mixture of drugs reaching lower dermatomes (T5 or below) (P < 0.001). Group M (Mixture group): Received 1.8 mL of 0.5% hyperbaric bupivacaine (9 mg) premixed with 0.3 mL fentanyl (15 mcg) as a single injection intrathecally. Group S (sequential group): Received 1.8 mL of 0.5% hyperbaric bupivacaine followed by 0.3 mL fentanyl in two syringes, injected sequentially at the same spinal puncture. T3: third thoracic dermatome; T4: fourth thoracic dermatome; T5: fifth thoracic dermatome; T6: sixth thoracic dermatome

Figure 5 depicts the maximum sensory level achieved. A higher proportion of Group S reached T4 or higher compared to Group M.

Rescue analgesia requirement

**Figure 6:**
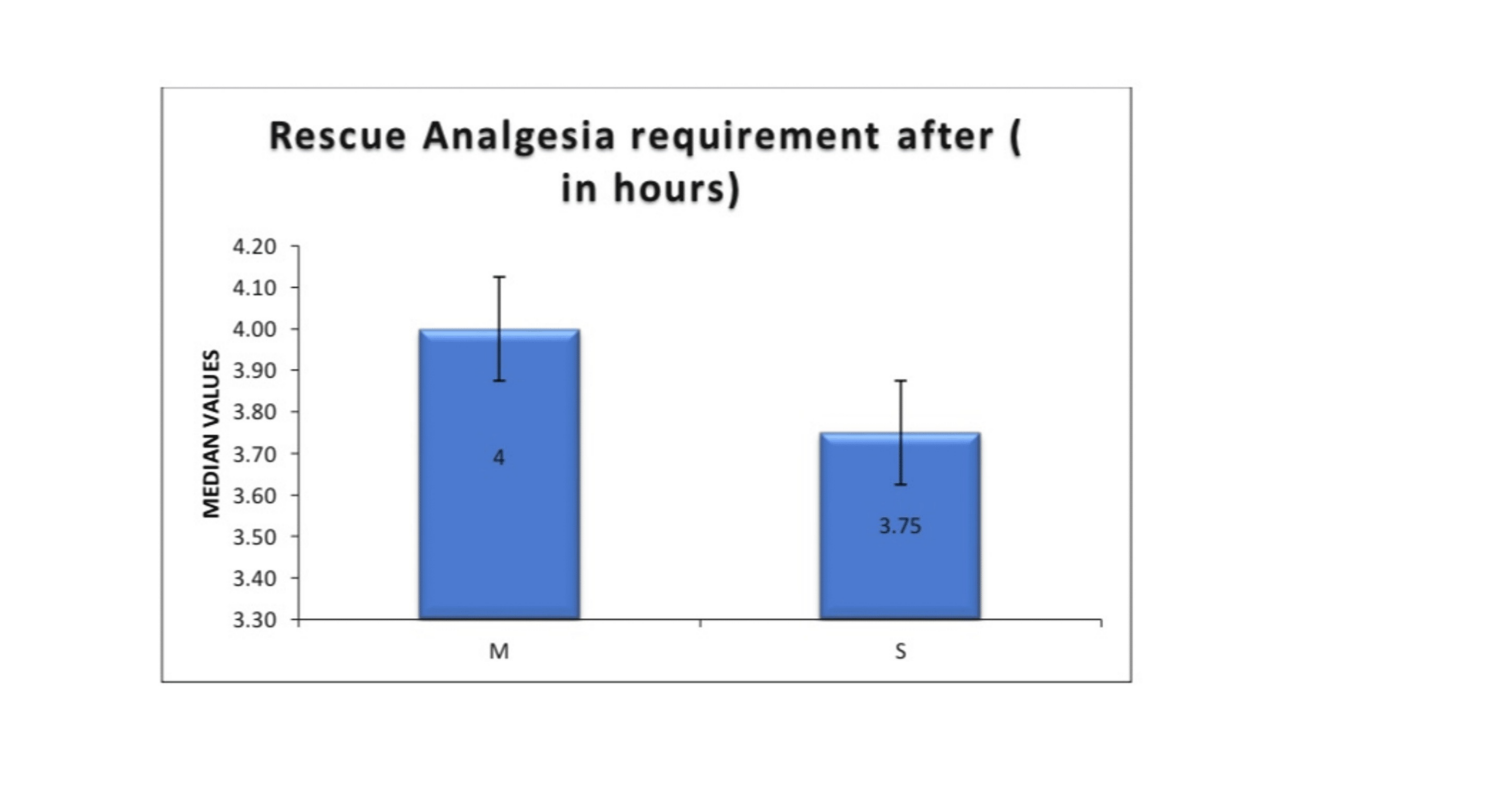
The time for the first rescue analgesia request between the two groups Group M (Mixture group): Received 1.8 mL of 0.5% hyperbaric bupivacaine (9 mg) premixed with 0.3 mL fentanyl (15 mcg) as a single injection intrathecally. Group S (Sequential group): Received 1.8 mL of 0.5% hyperbaric bupivacaine followed by 0.3 mL fentanyl in two syringes, injected sequentially at the same spinal puncture. Data are expressed as percentages.

The median time to first rescue analgesic request was similar between the two groups (Group M: 4 hours; Group S: 3.75 hours; P = 0.741) (Figure 6).

## Discussion

This randomized controlled trial demonstrated that sequential intrathecal administration of hyperbaric bupivacaine and fentanyl results in a significantly faster onset and prolonged duration of sensory and motor block compared to the conventional premixed approach in patients undergoing elective caesarean section.

The shorter time to achieve the T5 sensory level in the sequential group supports the concept that maintaining the native baricity of each agent enhances the cephalad spread of the anaesthetic within cerebrospinal fluid [7,8]. This is consistent with previous findings that show the physical properties of intrathecal solutions, such as baricity and density, critically influence their distribution in the subarachnoid space [7,8].

The improved block characteristics observed in the sequential group likely reflect enhanced pharmacodynamic interaction when the drugs are administered sequentially rather than combined, as reported in earlier studies [10]. The prolonged sensory and motor block durations are clinically beneficial in extending the pain-free interval postoperatively.

Although the differences in incidence of hypotension and vasopressor requirement were not statistically significant, the trend toward improved haemodynamic stability with the sequential technique aligns with earlier findings by Cesur et al. [9] and Keera and Elnabtity [11]. This effect may be attributed to the staggered sympathetic blockade produced by the sequential injection, which avoids the abrupt haemodynamic shifts often associated with premixed administration.

Importantly, the neonatal outcomes, including Apgar scores and umbilical cord blood gases, were comparable between groups, indicating that the sequential technique does not compromise fetal well-being. Similar safety outcomes have been reported by Desai et al. [12], who found no adverse neonatal effects from either method of administration.

Low-dose spinal anaesthesia has also been used to minimize hypotension, particularly in high-risk populations such as preeclamptic women [13,14]. Additionally, maternal positioning during spinal induction, such as sitting versus lateral, has been shown to influence anaesthetic spread and efficacy [15]. The use of additives like fentanyl in spinal anaesthesia is well-supported in the literature and enhances analgesia without compromising maternal or neonatal safety [16].

These findings affirm that the sequential administration technique is a safe, simple, and effective alternative for spinal anaesthesia in caesarean section, with potential benefits in clinical practice. Future studies involving larger, multicentre populations and evaluating long-term neonatal outcomes would be valuable in validating these results and exploring the broader applicability of this technique.

Hence, we concluded that the intrathecal injection of 9 mg of 0.5% hyperbaric bupivacaine followed by intrathecal injection of 15 mcg of fentanyl reduced the time to achieve complete sensory and motor block and delayed the regression of block and, being a sequential technique, does not have any effect on level of sedation and incidence of hypotension and vasopressor requirements or bradycardia as compared to the administration of mixed medications. The newborn’s outcome also remained unaffected.

Strengths and limitations

The strengths of the study include adequate sample size, rigorous randomisation, standardised anaesthetic and surgical protocols, and blinded outcome assessment. The limitations include the single-centre design, lack of long-term neonatal follow-up, and inability to blind the anaesthesiologist administering the drug due to the nature of the interventions.

Clinical implications

The sequential technique is simple and reproducible and offers enhanced block quality with a trend toward greater haemodynamic stability. It is particularly suitable for high-risk parturients or resource-limited settings.

Future directions

Multicentre trials with long-term neonatal outcome assessments are warranted. Comparative studies with other intrathecal adjuvants may further refine spinal anaesthesia techniques for obstetric surgery.

## Conclusions

The intrathecal injection of 9 mg of 0.5% hyperbaric bupivacaine followed by intrathecal injection of 15 mcg of fentanyl reduced the time to achieve complete sensory and motor block and delayed the regression of block and, being a sequential technique, does not have any effect on the level of sedation and incidence of hypotension and vasopressor requirements or bradycardia compared to the administration of mixed medications. The newborn’s outcome also remained unaffected.
